# Evaluation of in vitro and in vivo biocompatibility of a myo-inositol hexakisphosphate gelated polyaniline hydrogel in a rat model

**DOI:** 10.1038/srep23931

**Published:** 2016-04-13

**Authors:** Kwang-Hsiao Sun, Zhao Liu, Changjian Liu, Tong Yu, Tao Shang, Chen Huang, Min Zhou, Cheng Liu, Feng Ran, Yun Li, Yi Shi, Lijia Pan

**Affiliations:** 1 Department of Vascular Surgery, Nanjing Drum Tower Hospital, the Affiliated Hospital of Nanjing University Medical School, Nanjing, China; 2 Jiangsu Provincial Key Laboratory of Photonic and Electronic Materials, Collaborative Innovation Center of Advanced Microstructures, School of Electronic Science and Engineering, Nanjing University, Nanjing, China

## Abstract

Recent advances in understanding the interaction between electricity and cells/biomolecules have generated great interest in developing biocompatible electrically conductive materials. In this study, we investigated the biocompatibility of a myo-inositol hexakisphosphate gelated polyaniline hydrogel using *in vitro* and *in vivo* experiments in a rat model. The polyaniline hydrogel was used to coat a polycaprolactone scaffold and was cultured with rat endothelial progenitor cells differentiated from rat adipose-derived stem cells. Compared with the control sample on a pristine polycaprolactone scaffold, the treated polyaniline hydrogel had the same non-poisonous/cytotoxicity grade, enhanced cell adhesion, and a higher cell proliferation/growth rate. In implant studies, the polyaniline hydrogel sample induced milder inflammatory responses than did the control at the same time points. Combining the advantages of a biocompatible hydrogel and an organic conductor, the inositol phosphate-gelated polyaniline hydrogel could be used in bioelectronics applications such as biosensors, neural probes, cell stimulators, medical electrodes, tissue engineering, and electro-controlled drug delivery.

Interest in the development of biocompatible and conductive materials has increased in recent years because the interaction between electrical fields/current and tissues/cells/biomolecules can be used for several biological and medical applications^1^,^2^. In this field, conducting polymers show enormous potential for providing a mechanically soft and electrically conducive platform for cell integration. Conducting polymers have been extensively used as stimulators for contractile myotubes and neuron electrodes^3^,^4^, controllers of myoblast cell differentiation^5^, biosensors^6^, drug delivery agents^7^, tissue engineering devices^8^, and medical electrodes^9^. Furthermore, by combining the unique features of hydrogels, such as their high water content and a three-dimensional (3D) framework that is similar to the extracellular environment, conducting polymer hydrogels are emerging as the next generation of bioactive electrodes to improve both the interface between synthetic and biological systems and the performance of medical devices^10^,^11^,^12^,^13^,^14^.

Generally, biomedical applications call for simple and effective control over the desired properties of materials, such as high conductivity, biological compatibility, lack of cell toxicity, and lack of susceptibility to infection. Our group has proposed a highly conductive, high water content, and hierarchically nanostructured polyaniline (PAni) hydrogel^15^. The synthesis strategy is to use myo-inositol hexakisphosphate (IP6, also known as phytic acid; the molecular structure is shown in Scheme 1a) to gelate polyaniline and form a hydrogel^15^. IP6 is a good ionic conductor and a natural molecule that is widely available in plant tissues, such as bran and seeds^16^,^17^, and that affects a variety of cellular functions, such as signal transduction^17^, cell proliferation^18^, cell differentiation^19^, exocytosis^20^, antioxidants^21^, mRNA transport^22^, and DNA repair^23^. In this research, we investigated the *in vitro* and *in vivo* biocompatibility of the PAni hydrogel in a rat model. Compared to the control sample of a pristine polycaprolactone (PCL) scaffold, the IP6-gelated PAni hydrogel had greater *in vitro* and *in vivo* biocompatibility, including a same non-poisonous/cytotoxicity grade, enhanced cell adhesion and proliferation, and a lower inflammatory response.

## Results

### Materials

**Figure 1:**
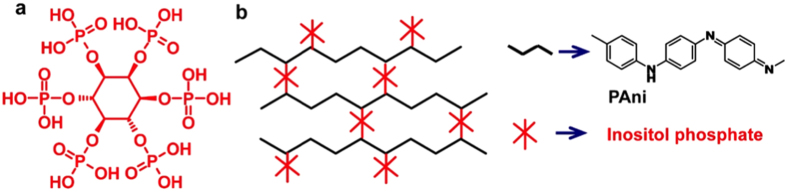
Schematic illustration of the chemical structure of the inositol phosphate-gelated PAni hydrogel. (**a**) Molecular structure of myo-inositol hexakisphosphate (IP6). (**b**) Mechanism for the IP6 gelation of the PAni hydrogel.

The PAni hydrogel was applied to the PCL scaffold by dip-coating the scaffold in a precursor solution. PCL scaffolds fabricated by the electrospinning method were selected as the substrates and the control group for the PAni hydrogel because PCL have good biocompatibility and excellent mechanical properties and they are biodegradable. The gelation mechanism of the PAni hydrogel is illustrated in Fig. 1. IP6 reacts with PAni by protonating the nitrogen groups on PAni. Because each IP6 molecule can interact with more than one PAni chain, this crosslinking effect results in the formation of a mesh-like hydrogel network composed of interconnected PAni chains (Fig. 1a). In a typical coating procedure, a solution containing the oxidative initiator (solution A) was mixed with a solution containing the aniline monomer and IP6 (solution B). Then, the mixed solution was used to dip-coat the PCL scaffold. PAni was polymerized and gelated in approximately 3 min, and the color of the substrate changed to dark green (the color of emeraldine PAni). The substrate was rinsed and purified with deionized (DI) water to remove excess ions. The PAni hydrogel has a conductivity of ~0.11 S cm^−1^ and a water content of approximately 92%. Thereafter, the PAni hydrogel-coated PCL scaffolds were used for *in vitro* and *in vivo* biocompatibility evaluations.

### Morphology of the coated thin film and durability evaluation

**Figure 2:**
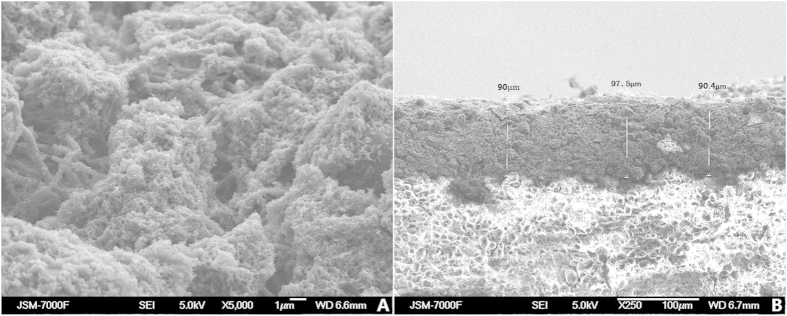
SEM images of the IP6-gelated PAni coating PCL scaffolds. (**A**) The surface morphology of the IP6-gelated PAni coated PCL scaffolds. (**B**) The cross section of the IP6-gelated PAni coating on PCL scaffolds.

The surface morphologies of the IP6-gelated PAni coated PCL scaffolds were showed as Fig. 2A. The SEM observation showed a nanostructured morphologies of the surface of IP6-gelated PAni coated PCL scaffolds and enough thickness for applications. The thickness of the IP6-gelated PAni coating on PCL scaffolds was about 90–100 μm showed as Fig. 2B. The PAni thin film has good adhesion on the PCL scaffolds that there wasn’t any macro obscission observed after the samples were soaking, washing, and wiped by alcohol cotton ball. FTIR spectroscopy was used to investigate the surface characteristics of the substrate before and after coating as presented in Supplementary Fig. S1. Supplementary Fig. S1A was the result of blank PCL scaffold. Supplementary Fig. S1B was the result of PAni-coated PCL scaffolds before soaking and washing. Supplementary Fig. S1C–E presents the result of PAni-coated PCL scaffolds after 1, 5 and 7 days soaking and washing, respectively. The FTIR results of samples after soaking and washing (Supplementary Fig. S1C–E) showed a high similarity with the pristine samples (Supplementary Fig. S1B).

### Seed cell differentiation and cytotoxicity testing-MTT assay

Rat endothelial progenitor cells (rEPCs) differentiated from rat adipose-derived stem cells (rADSCs) were used as seed cells in the *in vitro* experiments. After 3–5 passages of the primary culture, the morphology of rEPCs was regular. Immunostaining demonstrated the presence of CD34, CD133 and VWF (Supplementary Fig. S2). These results confirmed that differentiated rEPCs were obtained.

Cytotoxicity testing using the MTT assay is a primary indicator in the bio-evaluation of medical devices. rEPCs were grown in 96-well culture plates with the corresponding extract solution (PAni-coated PCL scaffolds (PCPS), blank PCL scaffolds (BPS)) or EGM-2 (negative control (NC) group). The optical density (OD) values for each group were measured after cultivation for 1, 5 and 7 days. The OD values were used to calculate the relative growth rate (RGR) using the formula listed in the preceding paragraphs, and the cytotoxicity grade was assessed according to the cytotoxicity grading criteria shown in Table 1^24^. The OD values and the RGRs of each group at different time points are shown in Table 2. There were significant (P < 0.05) difference between the same sample group with different time points and the same time point with different sample groups. The RGR of rEPCs in the extract solution of the PCPS group was 160% (1 day), 118% (5 days) and 130% (7 days). The RGR of the BPS group was 160% (1 day), 104% (5 days) and 129% (7 days). Compared with the BPS group, the PCPS group had the same or higher RGR at the same time points. In addition, as shown in Table 2, both groups had a cytotoxicity grade of 0 throughout the cultivation process.

### Cell morphology on scaffolds

**Figure 3:**
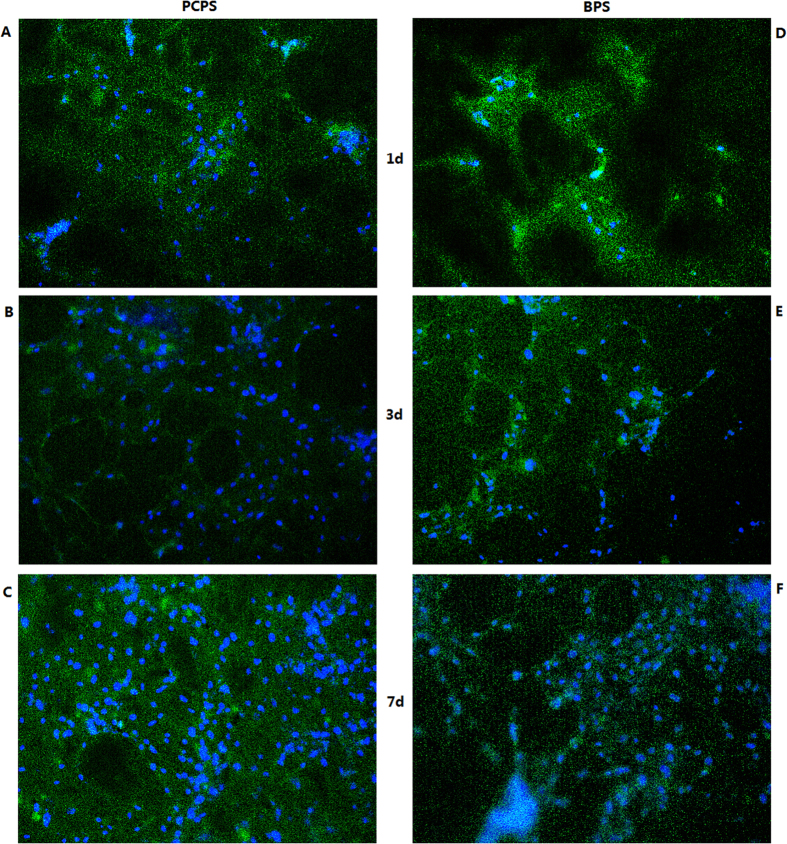
Immunofluorescence images of rEPCs seeded on the PCPS and BPS surfaces and then cultured for 1, 3 and 7 days (VWF positive staining) (×200). (**A**) PCPS (1 day), (**B**) PCPS (3 days), (**C**) PCPS (7 days), (**D**) BPS (1 day), (**E**) BPS (3 days), and (**F**) BPS (7 days).

**Figure 4:**
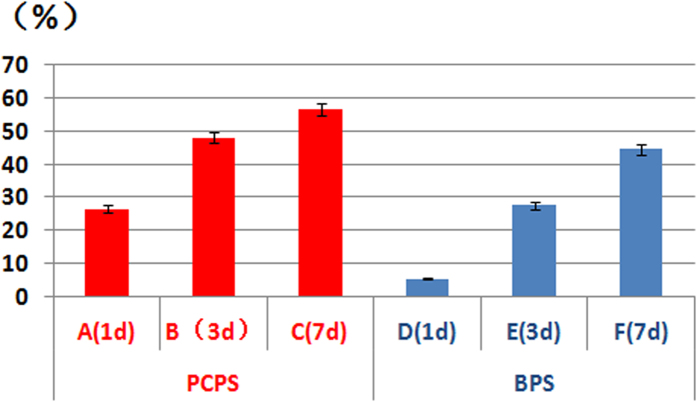
Area specific cell density on the PCPS and BPS surfaces observed by immunofluorescence. (A) PCPS (1 day), (B) PCPS (3 days), (C) PCPS (7 days), (D) BPS (1 day), (E) BPS (3 days), and (F) BPS (7 days).

Immunofluorescence microscopy and scanning electron microscopy (SEM) were used to investigate the morphology of the rEPCs that grew on the PCPS and BPS surfaces. The immunofluorescence images are presented in Fig. 3. The quantity of cells on the surfaces of the scaffolds increased with the duration of cultivation in the same group. Notably, compared with the BPS group (Fig. 3D–F), the PCPS group (Fig. 3A–C) exhibited greatly enhanced adhesion and proliferation. The area specific cell density (defined as the area covered with cells relative to the surface area of the substrate) values were 25–28% vs. 5–6% on day 1, 45–51% vs. 26–29% on day 3, and 53–60% vs. 42–47% on day 7 for the PCPS groups vs. the BPS group (Fig. 4).

**Figure 5:**
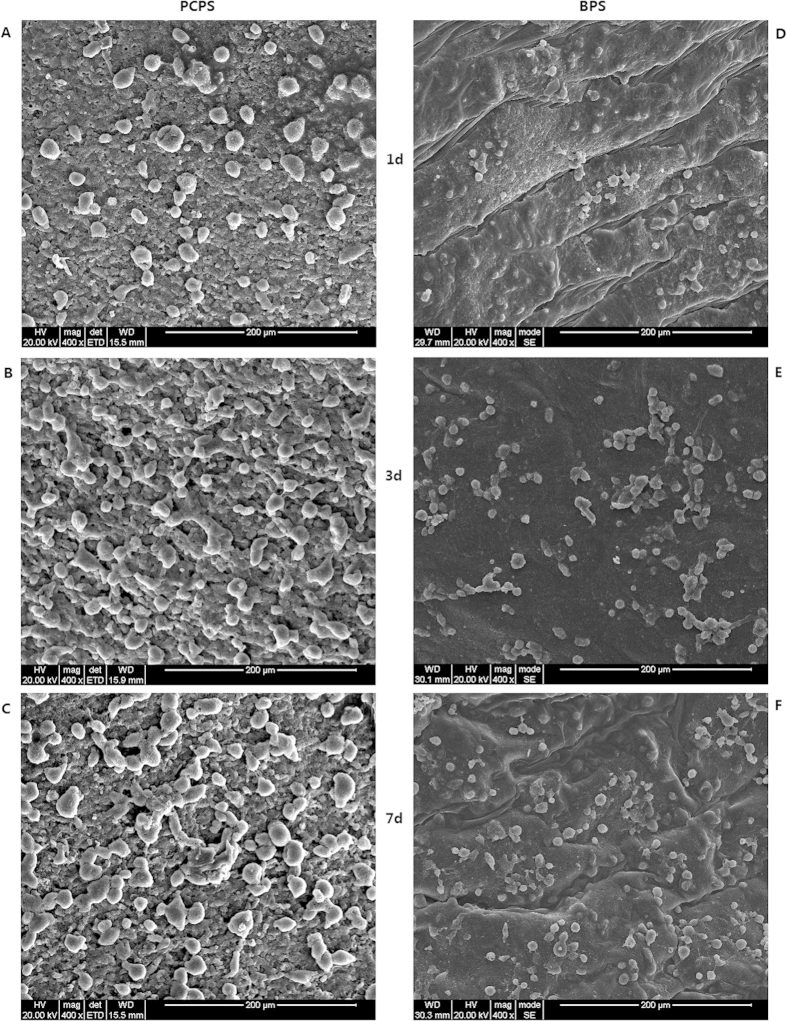
SEM images of rEPCs seeded on the PCPS and BPS surfaces and then cultured for 1, 3 and 7 days. (**A**) PCPS (1 day), (**B**) PCPS (3 days), (**C**) PCPS (7 days), (**D**) BPS (1 day), (**E**) BPS (3 days), and (**F**) BPS (7 days).

**Figure 6:**
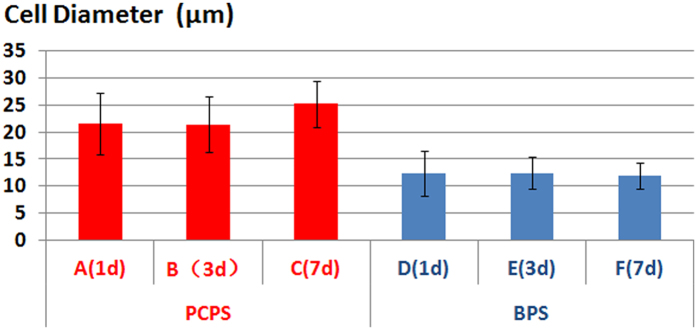
Average diameter of cells after seeding and culturing on the PCPS and BPS surfaces. (A) PCPS (1 day), (B) PCPS (3 days), (C) PCPS (7 days), (D) BPS (1 day), (E) BPS (3 days), and (F) BPS (7 days).

**Figure 7:**
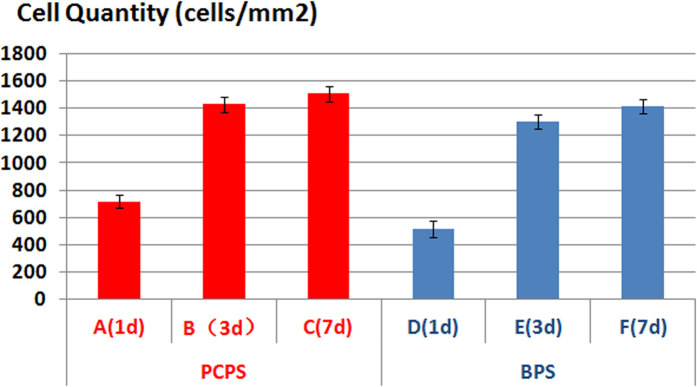
Cell quantity in unit area after seeding and culturing on the PCPS and BPS surfaces. (A) PCPS (1 day), (B) PCPS (3 days), (C) PCPS (7 days), (D) BPS (1 day), (E) BPS (3 days), and (F) BPS (7 days).

SEM images of rEPCs grown on the PCPS and BPS surfaces are shown in Fig. 5. At each time point, the PCPS group exhibited better cell adhesion, development and proliferation (Fig. 5A–C) than the BPS group (Fig. 5D–F). The cells grown on PCPS (Fig. 5A–C) were plump and had a much larger cell diameter compared with that of the BPS group (Fig. 5D–F). The average cell diameters of the PCPS and BPS groups are presented in Fig. 6. The average cell diameters of the PCPS and BPS groups were 21.52 μm vs. 12.35 μm (1 day), 21.37 μm vs. 12.45 μm (3 days) and 25.16 μm vs. 11.85 μm (7 days), respectively. The quantity of cells in unit area of the PCPS and BPS group are presented in Fig. 7. The quantity of cells in unit area of the PCPS and BPS group were 718 cells/mm^2^ vs. 513 cells/mm^2^ (1 day), 1428 cells/mm^2^ vs. 1300 cells/mm^2^ (3 day) and 1505 cells/mm^2^ vs. 1411 cells/mm^2^ (7 day), respectively.

### *In vivo* biocompatibility evaluation

The PCPS and BPS substrates were implanted in the backs of rats. The rats were sacrificed at specific time points (1, 2, and 4 weeks) after surgery. No behavioral changes or visible signs of physical impairment indicating systemic or neurological toxicity were observed during post-operative examinations or at the time of death. Gross observation of the implanted scaffolds at the time of death revealed that both PCPS and BPS were surrounded by fibrous tissue (Supplementary Fig. S4).

**Figure 8:**
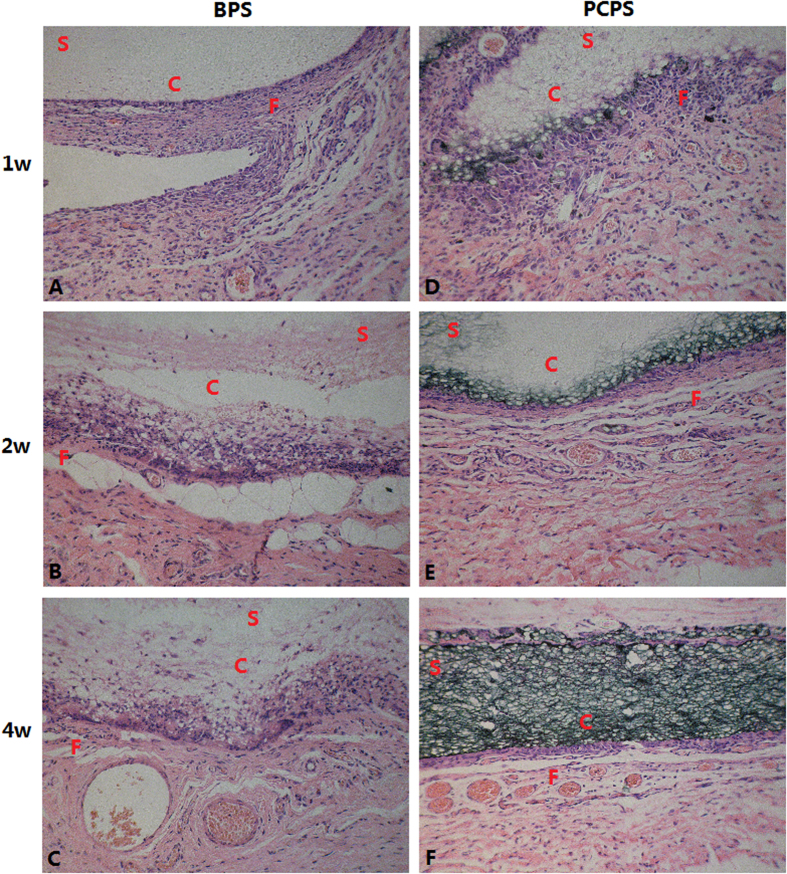
Micrographs of rat subcutaneous tissue responses to BPS and PCPS after different implantation times (×200). **(A)** BPS 1 week, **(B)** BPS 2 weeks, **(C)** BPS 4 weeks, **(D)** PCPS 1 week, **(E)** PCPS 2 weeks, and **(F)** PCPS 4 weeks. C = Scaffold implant site, F = Fibrous tissue, S = Scaffold.

The histological response induced by these scaffolds is summarized in Table 3. The inflammatory responses were graded as minimal, mild or moderate. The inflammatory responses of both the BPS and PCPS groups decreased with the time after implantation. In addition, the PCPS group had better histological responses than that of the BPS group. Detailed microscopic histological observations of the PCPS and BPS groups at specific time points are summarized below. 1 week after implantation, BPS evoked a moderate local inflammatory reaction characterized by the infiltration of lymphocytes, mononuclear macrophages and neutrophils and the formation of granulomas and multinucleated giant cells (Fig. 8A). PCPS also evoked a moderate local inflammatory reaction characterized by the infiltration of lymphocytes, mononuclear macrophages and neutrophils and by the formation of multinucleated giant cells (Fig. 8D). However, granulomas formed in the BPS group, but it not formed in the PCPS group, indicating a better histological response to PCPS than BPS at 1 week.

At 2 weeks, BPS elicited a mild to moderate local inflammatory response characterized by moderate or greater infiltration of lymphocytes, with the formation of multinucleated giant cells and fibrous tissue hyperplasia (Fig. 8B). PCPS evoked a mild local inflammatory response characterized by the moderate infiltration of lymphocytes, along with the formation of multinucleated giant cells and hyperplasia of fibrous tissue (Fig. 8E). The PCPS group had a relatively mild inflammatory response compared to that of the BPS group (mild vs. mild to moderate), as evaluated via the decreased infiltration of lymphocytes (moderate infiltration of lymphocytes vs. moderate to greater infiltration of lymphocytes).

At 4 weeks, both BPS (Fig. 8C) and PCPS (Fig. 8F) produced minimal inflammation characterized by sparse lymphocytic infiltration, with the formation of multinucleated giant cells and fibrous tissue proliferation. At this time point, the histological responses to PCPS and BPS were similar.

## Discussion

Generally, the term biocompatibility refers to the ability of a material that contacts living organisms/tissues to cause an appropriate host response^25^. The clinical safety of a medical substance and medical devices can be evaluated by determining the potential toxicity resulting from their contact with cells and tissues. Rational designs have improved the biocompatibility of conducting polymers in recent years; these techniques include surface modification^26^, combinations with hydrogels^12^ and the use of electrospun nanofibers^27^. However, most of these methods introduce insulating components into the conducting polymer matrix to enhance the biocompatibility of the conducting polymer while maintaining high conductivity. This requirement remains a challenge. Our group recently proposed a multi-functional doping acid crosslinking technique to gelate conducting polymer hydrogels^15^. In this work, a natural molecule, IP6, which is present at concentrations ranging from 10–100 μM in mammalian cells^19^, was used as the dopant acid to gelate PAni. Because IP6 itself is a good ionic conductor, the IP6-gelated PAni hydrogel exhibited a high conductivity of 0.11 S·cm^−1^.

The PAni hydrogel exhibited better *in vivo* and *in vitro* biocompatibility than the study control (electrospun PCL scaffolds). In this study, electrospun PCL was used as both the scaffolds of IP6-gelated PAni hydrogel and the control sample, because of the excellent mechanical properties and bio-degradability of PCL^28^. PCL has been demonstrated to have good biocompatibility^28^ and has been used in a number of biomedical applications^29^. The SEM observation (Fig. 2) showed a well morphologies on the surface of IP6-gelated PAni coated PCL scaffolds and enough thickness for applications. None macro obscission observed and high similar FTIR profiles between the samples after (Supplementary Fig. S1C–E) and before (Supplementary Fig. S1B) soaking and washing indicating the well stability of the IP6-gelated PAni coating on the surface. The RGR and the corresponding cytotoxicity grades of PCPS and BPS are shown in Table 2. Throughout the cultivation process, the RGR of both groups was ≥100%, and the corresponding cytotoxicity grade of both groups was 0. These results indicate that both PCPS and BPS are non-poisonous according to the definition of the United States Pharmacopeia^24^. Furthermore, the RGR of the PCPS group was the same as or higher than that of the BPS group at the same time point, indicating that PCPS had at least the same biocompatibility with BPS.

Immunofluorescence and SEM observation of cellular scaffolds revealed that the quantity of cells increased with the culture duration (Fig. 3–4) and that cells seeded on the PCPS surface had better adhesion and proliferation than those seeded on BPS (Fig. 5–7) at the same time point. The quantity of cells in unit area of the PCPS was more than BPS, the cells grown on PCPS were plumper, and the average diameter of the cells was larger by a factor of 1 than that of the BPS group. The better adhesion and proliferation of cells on PCPS indicated that the IP6-gelated PAni hydrogel had better biocompatibility than the PCL scaffold, most likely due to the improved surface properties of IP6-gelated PAni. In particular, the water content of the PAni hydrogel was as high as ~92%. Moreover, IP6-gelated PAni has excellent hydrophillicity, with a water contact angle ~24° compared with ~69° for pristine PAni.

The implant experiments also suggested that the IP6-gelated PAni hydrogel had better *in vivo* biocompatibility than that of the PCL scaffold. BPS and PCPS were implanted in the backs of rats, and the rats were sacrificed at specific time points (1, 2, and 4 weeks) after surgery to observe the histological response of scaffolds using light microscopy. As shown in Table 3 and Fig. 8, a decreasing inflammatory response with the duration of time after implantation was apparent in both the BPS and PCPS groups; the PCPS group exhibited an inflammatory response (Fig. 8D–F) that was milder than (1 week and 2 week) or similar to (4 week) the response to BPS (Fig. 8A–C) at each time point.

**Figure 9:**
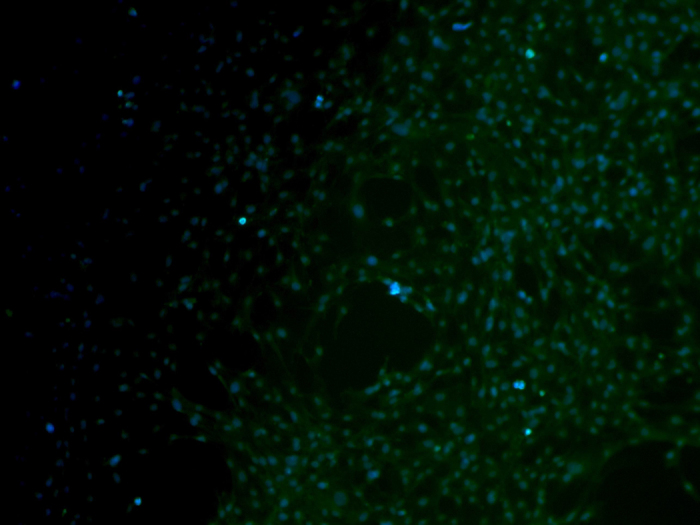
Immunofluorescence images of rat smooth muscle cells seeded on the IP6-gelated PAni coated substrates (×100).

The IP6-gelated PAni hydrogel has good biocompatibility with cells other than rEPCs, we has successively cultured the rat smooth muscle cell on this substrate (Fig. 9).

In summary, a natural molecule, IP6, was used to gelate a PAni hydrogel. The SEM and FTIR observation indicated that the IP6-gelated PAni coating on the surface of scaffolds had nanostructured morphologies, enough thickness and good stability. The results of cytotoxicity testing, immunofluorescence, cell morphology observation by SEM, and implant study experiments indicated that the IP6-gelated PAni hydrogel exhibited excellent *in vitro* and *in vivo* biocompatibility compared to that of the control (pristine electrospun PCL scaffold). We envision that this type of biocompatible and conductive PAni hydrogel will have great potential for future biomedical applications, including biosensors, bio-actuators, neural probes, tissue engineering, cell stimulation, DNA synthesis, and drug delivery.

## Methods

The study protocols were approved by the Ethics Committee of Nanjing Drum Tower Hospital, the Affiliated Hospital of Nanjing University Medical School. All experiments were performed in accordance with relevant guidelines and regulations.

### Prosthesis preparation

The PCL scaffolds were prepared by electrospinning a solution of 15% PCL in DMF on the FM-11 system (Future Material Sci-tech Co., Ltd., Beijing, China). The spinning conditions were as follows: 20 cm tip-collector distances, 20 kV voltage, 4 ml/h flow rate, and 2 h spin time. The typical electrospinning process was as follows. The high-voltage positive electrode was connected to the needle, and the negative electrode was connected to the collector. Afterwards, the prepared PCL solution was ejected by pumping through a sterilized syringe and a needle. Owing to the high voltage of the electrodes, there was a constant electric field between the needle and collector, which permitted the solution to overcome the surface tension and escape from the needle tip. During the escape process, the solvent evaporated, and fibers formed. These fibers were collected by the collector and formed into a PCL scaffold. The PCL scaffolds were cut from the collector using a surgical scalpel and were dried under reduced pressure at 37 °C for 24 h.

After fabricating the PCL scaffolds, we used IP6-gelated PAni to coat the scaffolds. In the first step, the precursor solutions were prepared: Solution A, an aqueous solution of ammonium persulfate, and Solution B, an aqueous solution of aniline monomer and phytic acid. In the second step, solutions A and B were mixed, and the mixed solution was used to dip-coat the PCL scaffolds. The PAni precursor solution usually gelated within 3 min. In the third step, the sample was placed in deionized water for 2 h for depuration.

Finally, both the PAni-coated PCL scaffolds (PCPS) and blank PCL scaffolds (BPS) were sterilized by gamma irradiation (25 kGy) before the *in vitro* and *in vivo* experiments. The surface morphologies of the IP6-gelated PAni coated PCL scaffolds and thickness of the IP6-gelated PAni coating on PCL scaffolds were observed by a field emission scanning electron microscope (FESEM; model JSM-7000F; JEOL Ltd, Tokyo, Japan).

### Durability investigation

The durability of the hydrogel film was studied by mechanical abrasion and FTIR spectroscopy. Fourier-transform infrared spectroscopy (FTIR) measurements were performed using a NEXUS-870 FTIR spectrometer (Nicolet, USA). Scaffolds with IP6-gelated PAni coating were used for stability studies as described previously^30^. The scaffolds were soaked in 1 mL of water for 1, 5 and 7 days. Then, the substrates were dipping and swirling in 10 mL water for 5 times. The process was repeated 3 times, each time in fresh water. FTIR spectra of the scaffolds before and after the soaking were recorded. The blank PCL scaffold was used as a control. Samples were dried after washing prior to the recording of the spectra. And before and after soaking and washing, the coated samples were wiped softly by alcohol cotton ball for observing the stability of the caoting with the macro perspective.

### Seed cell preparation

We harvested rADSCs from adult male Sprague–Dawley rats (weighing 350–400 g) purchased from the Jiesijie Laboratory Animal Co., Ltd. [Shanghai, China, license no. SCXK (hu) 2013-0006] as described previously^31^. The rats were sacrificed by cervical dislocation and immersed in 75% ethanol for 1 h. Then, the inguinal adipose tissue of rats was obtained and washed with equal volumes of PBS to remove the blood cells under aseptic conditions. The adipose tissue was digested with 0.1% collagenase type I (Sigma-Aldrich, St. Louis, MO, USA) with intermittent shaking at 37 °C for 1 h. Then, the floating adipocytes were separated from the stromal cell fraction via centrifugation (1,200 rpm for 5 min). After centrifugation, the cell suspension was filtered through a 100 μm nylon mesh (BD Biosciences, San Jose, CA, USA). After re-suspension, the harvested rADSCs were cultured in basal medium (Lonza Inc., Walkersville, MD, USA) containing 10% fetal bovine serum (Gibco, Gaithersburg, MD, USA) at 37 °C with 5% CO_2_. The medium was changed the next day to remove the non-adherent cells. Afterwards, the medium was changed every 2 days.

To obtain rEPCs for seed cells, rADSCs with EGM-2 (Lonza Inc., Walkersville, MD, USA) were incubated at 37 °C with 5% CO_2_ in air. Afterwards, the medium was changed every 2 days. Differentiated rEPCs were identified via immunofluorescence.

### Immunofluorescence

The differentiated rEPCs were seeded on 25-mm glass-bottom dishes and then immersed in EGM-2 medium overnight. Before immunostaining, the cells were fixed with 4% paraformaldehyde for 15 min and permeabilized in 0.5% Triton X-100 for 20 min at room temperature. Afterwards, the cells were incubated with primary antibody at 4 °C overnight. The primary antibodies included CD34 (PAB18289, Abnova, Taipei, Taiwan), CD133 (PL030395R, PL Laboratories, British Columbia, Canada), VWF (ab6994, Abcam, Cambridge, UK), and IgG (ab6730, Abcam, Cambridge, UK). The cells incubated with IgG served as the negative control. Subsequently, the cells were incubated with FITC fluorescent secondary antibody (bs-0295G-FITC, Biosynthesis Biotechnology Co., Ltd. Beijing, China) at 37 °C for 1 h. Thereafter, the cells were incubated with DAPI (Beyotime Institute of Biotechnology, Shanghai, China) and visualized by fluorescence microscopy (AXIO SCOPE A1, Zeiss, Germany).

### Cytotoxicity testing-MTT assay

The cytotoxicity of the PAni-coated PCL scaffolds and blank PCL scaffolds was evaluated *in vitro* using rEPCs via the MTT colorimetric assay. Three groups were tested: (1) PCPS; (2) BPS; and (3) NC. In the PCPS and BPS groups, the rEPCs were grown in 96-well culture plates at a density of 5 × 10^3^ cells per well with the appropriate extract solution for each group. We prepared the extract solution of each group according to the protocol outlined in ISO 10993^32^. In the NC group, the rEPCs were dispensed at the same density via EGM-2. Then, the cells were cultured in humidified 95% air/5% CO_2_ at 37 °C. The extract solution and medium were changed every 2 days. The observation periods were 1 day, 5 days and 7 days. At the end of the observation period, 20 μl of MTT (5 mg/ml) was added to each well, and the plates were incubated at 37 °C for another 4 h. Afterwards, 150 μl dimethyl sulfoxide (DMSO) was added to each well; the wells were then gently shaken for 10 min to dissolve the formazan crystals. The optical density (OD) values were measured at 490 nm by a microplate reader (ELX800, BioTek, Winooski, VT).

The cytotoxicity of the material was assessed via the RGR of cells according to the United States Pharmacopeia^24^. The RGR was calculated using the formula RGR = (OD value of samples)/(OD value of negative control). The cytotoxicity grading criteria are listed in Table 1.

Cytotoxicity results are expressed as means ± standard deviation for n = 6. Uptake data were analyzed using SPSS version 17.0 (SPSS Inc., Chicago, IL, USA). Statistically significant difference was considered at P < 0.05.

### Cell seeding

There were 2 groups in this test: (1) PCPS and (2) BPS. To observe the *in vitro* adhesion and morphology of both groups, we cut both PCPS and BPS samples into circles with diameters of 20 mm and sterilized them via gamma irradiation (25 kGy). Afterwards, the rEPCs were trypsinized and seeded on the surface of each scaffold, which were then placed in 12-well culture plates. Each scaffold was seeded with 1 × 10^6^ cells, followed by the addition of 3 ml of EGM-2 using thin glass rings adapted to the inner diameter of the wells to prevent the scaffolds from floating. Subsequently, the cellular scaffolds were cultured at 37 °C with 5% CO_2_ in air. The medium was changed every 2 days. Afterwards, the cell morphology of each group was observed by SEM and immunofluorescence after 1, 3 and 7 days of culture.

### Immunofluorescence of the scaffolds

After cultivation for 1, 3 and 7 days, the cellular scaffolds of both groups were harvested and washed with PBS to remove the non-adherent cells. Afterwards, the scaffolds were fixed in 4% paraformaldehyde for 15 min at room temperature followed by permeation with 0.5% Triton X-100 for 20 min. The samples were incubated in primary antibody overnight at 4 °C. The primary antibody was VWF. Subsequently, the samples were incubated in the FITC fluorescent secondary antibody for 1 h at 37 °C. Finally, we washed and incubated the cells with DAPI before visualization by fluorescence microscopy (AXIO Observer A1, Zeiss, Germany).

### Scanning electron microscopy of the scaffolds

After cultivation for 1, 3 and 7 days, the cellular scaffolds were harvested and washed with PBS to remove the non-adherent cells. Afterwards, the scaffolds were fixed in 2.5% glutaraldehyde for 24 h at room temperature. The samples were fixed with 1% osmium tetroxide for ~2–4 h and then dehydrated with a graded ethanol series (from 30 to 100% in steps of 20% for 10 min each). After dehydration, the samples were lyophilized for 3 days. All samples were sputter-coated with gold before SEM observation, which was performed using a FEI Quanta 200 scanning electron microscope (FEI Company, Hillsboro, USA). SEM images of both PAni-coated PCL scaffolds and blank PCL scaffolds were obtained at an accelerating voltage of 20 kV.

### Implant studies

Male Sprague-Dawley rats (weighing 350–400 g) were purchased from the Jiesijie Laboratory Animal Co., Ltd. [Shanghai, China, license no. SCXK (hu) 2013-0006] and fed standard pellets and water ad libitum. There were 30 rats used for implantation (each time point of each groups with 5 rats), and each rat implanted with 2 scaffolds. Before implantation, both PCPS and BPS scaffolds were cut into squares (approximately 6 mm × 6 mm) and sterilized by gamma irradiation (25 kGy). The surgical instruments were sterilized by autoclaving. The rats were anaesthetized with 10% chloral hydrate (0.3 ml/100 g, i.p.) prior to the operation. Then, the hair on the rat dorsum was shaved and sterilized with 75% ethanol and propidium iodide. An incision (approximately 2.5 cm long) on each side of the dorsum was made using a scalpel. Next, subcutaneous pouches were created in the incisions using blunt scissors, and scaffolds were implanted into each pocket (Supplementary Fig. S3). Upon implantation of the scaffold into the pouch, the wounds were sutured with 3/0 Mersilk^®^ sutures (Ethicon, Johnson & Johnson Medical Ltd, UK) (Supplementary Fig. S5).

The rats were sacrificed by cervical dislocation at specific time points (1, 2, and 4 weeks) after surgery. The tissues associated with and adjacent to the scaffolds were harvested using a scalpel. The scaffolds with the surrounding tissue were fixed in 4% paraformaldehyde for histology.

Prior to being embedded in paraffin wax, the samples were dehydrated in a Vacuum Infiltration Processor (Tissue-Tek^®^VIP^®^ 5, Sakura Fineterk, Torrance, CA) using a graded series of alcohol. Then, the samples were embedded in paraffin using an embedding machine (Tissue-Tek^®^ TEC™ 5, Sakura Fineterk, Torrance, CA), sectioned using a microtome (Leica RM2016, Leica Microsystems, Germany), and stained with hematoxylin and eosin. The samples were viewed using light microscopy by an independent pathologist who observed the inflammatory responses in the region around the scaffolds. Tissue inflammatory responses were evaluated as minimal, mild, or moderate according to the evaluation system described previously^33^,^34^.

## Additional Information

**How to cite this article**: Sun, K.-H. *et al.* Evaluation of in vitro and in vivo biocompatibility of a myo-inositol hexakisphosphate gelated polyaniline hydrogel in a rat model. *Sci. Rep.* **6**, 23931; doi: 10.1038/srep23931 (2016).

## Supplementary Material

Supplementary Information

## Figures and Tables

**Table 1:** Cytotoxicity grading criteria.

RGR (%)	Cytotoxicity Grade
100+	0 (non-poisonous, qualification)
75–99	1 (slightly poisonous, qualification)
50–74	2 (moderately poisonous, disqualification)
25–49	3 (severely poisonous, disqualification)
1–24	4 (disqualification)
0	5 (disqualification)

**Table 2:** OD value, relative growth rate (RGR) of extract solutions (n = 6, means ± standard deviation).

Samples	Culture period (days)	OD (x ± s)	RGR (%)	Cytotoxicity Grade
**PCPS**	1	0.096 ± 0.029	160	0
	5	0.492 ± 0.021	118	0
	7	0.598 ± 0.021	130	0
**BPS**	1	0.096 ± 0.020	160	0
	5	0.433 ± 0.074	104	0
	7	0.593 ± 0.045	129	0
**NC**	1	0.060 ± 0.032		
	5	0.416 ± 0.060		
	7	0.460 ± 0.018		

Between the same sample group with different time points, P < 0.05; Between the same time point with different sample groups, P < 0.05.

**Table 3:** Histological response of implanted scaffolds.

	1 week	2 weeks	4 weeks
BPS	MOD	MILD to MOD	MIN
PCPS	MOD	MILD	MIN

MIN: minimal; MILD: mild; MOD: moderate.
