# Supplementary material for: Evaluation of in vitro and in vivo biocompatibility of a myo-inositol hexakisphosphate gelated polyaniline hydrogel in a rat model
Source: Sci Rep. 2016 Apr 13;6:23931. doi: 10.1038/srep23931 (PMC4829851; doi:10.1038/srep23931)
Supplement: Supplementary Information [file srep23931-s1.doc]

**Evaluation of in vitro and in vivo biocompatibility of a myo-inositol hexakisphosphate gelated polyaniline hydrogel in a rat model**

Kwang-Hsiao Sun^1^, Zhao Liu^1*^, Changjian Liu^1^, Tong Yu^1^, Tao Shang^1^, Chen Huang^1^, Min Zhou^1^, Cheng Liu^1^, Feng Ran^1^, Yun Li^2^, Yi Shi^2^, Lijia Pan^2*^

^1^Department of Vascular Surgery, Nanjing Drum Tower Hospital, the Affiliated Hospital of Nanjing University Medical School, Nanjing, China

^2^Jiangsu Provincial Key Laboratory of Photonic and Electronic Materials, Collaborative Innovation Center of Advanced Microstructures, School of Electronic Science and Engineering, Nanjing University, Nanjing, China

*To whom correspondence should be addressed.

Email: (Z. L.) liuzhao83@gmail.com;

(L. P.) ljpan@nju.edu.cn

**Materials.** PCL (Mn 80,000) (Product Number: 440744), Aniline (ACS reagent, ≥99.5%) (Product Number: 242284), ammonium persulfate (AR, ≥98.0%) (Product Number: A3678), and phytic acid (50 %, w/w in water) (Product Number: 593648) were purchased from Sigma-Aldrich. N, N-Dimethylformamide (DMF) of analytical grade was purchased from Kelong Chemical Co., Ltd. (Chengdu, China). All products were used as received.


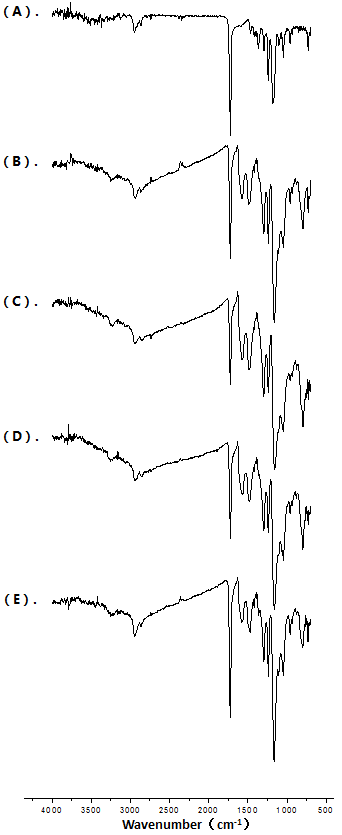


**Figure S1 | FTIR profiles of the different surfaces.** A: Blank PCL scaffold; B: PAni-coated PCL scaffolds before soaking and washing; C: PAni-coated PCL scaffolds after soaking and washing (1 day); D: PAni-coated PCL scaffolds after soaking and washing (5 days); E: PAni-coated PCL scaffolds after soaking and washing (7 days).


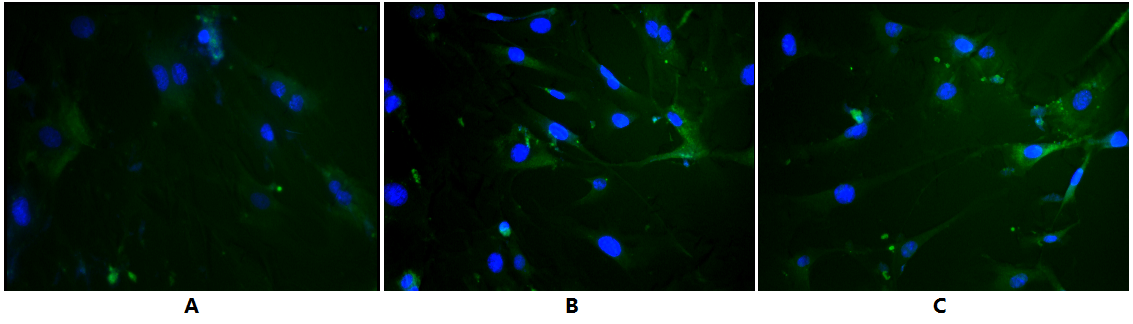


**Figure S2 | The rEPCs used as seed cell showed positive staining for CD34 (A), CD133 (B) and VWF (C) (× 200).**


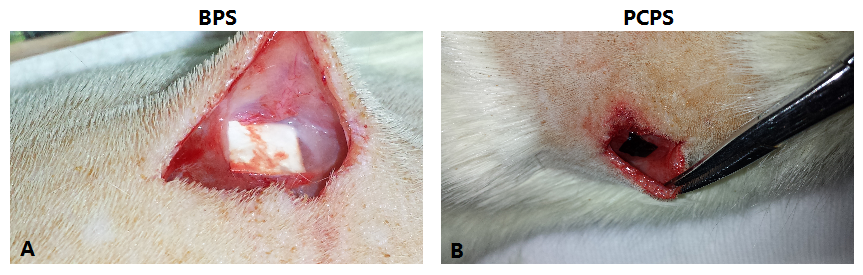


**Figure S3 | Implantation of BPS (A) and PCPS (B).**


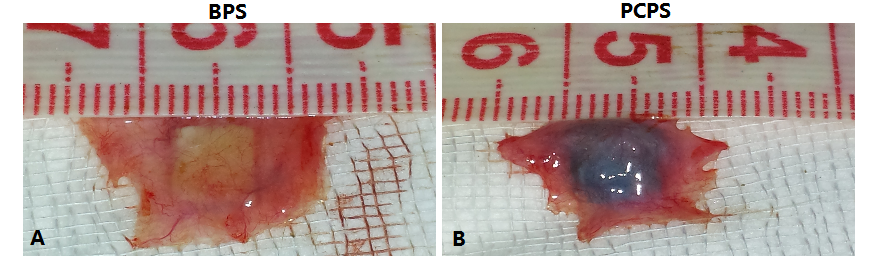


**Figure S4 | BPS (A) and PCPS (B) with surrounding tissue.**


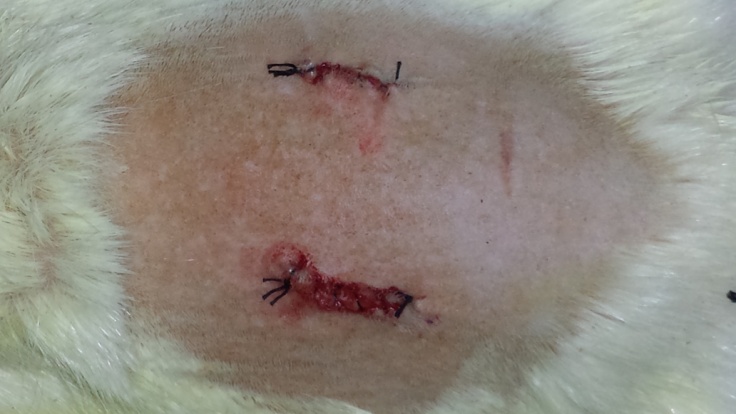


**Figure S5 | Suturation of wounds on the dorsum.**
